# Modulation of learning safety signals by acute stress: paraventricular thalamus and prefrontal inhibition

**DOI:** 10.1038/s41386-023-01790-2

**Published:** 2024-01-05

**Authors:** Zongliang Wang, Zeyi Wang, Qiang Zhou

**Affiliations:** State Key Laboratory of Chemical Oncogenomics, Guangdong Provincial Key Laboratory of Chemical Genomics, Peking University Shenzhen Graduate School, Shenzhen, 518055 China

**Keywords:** Prefrontal cortex, Fear conditioning

## Abstract

Distinguishing between cues predicting safety and danger is crucial for survival. Impaired learning of safety cues is a central characteristic of anxiety-related disorders. Despite recent advances in dissecting the neural circuitry underlying the formation and extinction of conditioned fear, the neuronal basis mediating safety learning remains elusive. Here, we showed that safety learning reduces the responses of paraventricular thalamus (PVT) neurons to safety cues, while activation of these neurons controls both the formation and expression of safety memory. Additionally, the PVT preferentially activates prefrontal cortex somatostatin interneurons (SOM-INs), which subsequently inhibit parvalbumin interneurons (PV-INs) to modulate safety memory. Importantly, we demonstrate that acute stress impairs the expression of safety learning, and this impairment can be mitigated when the PVT is inhibited, indicating PVT mediates the stress effect. Altogether, our findings provide insights into the mechanism by which acute stress modulates safety learning.

## Introduction

Safety learning is a process that associates a stimulus with the absence of threats, a topic of growing interest in behavioral research, yet its underlying mechanisms remain inadequately elucidated [1, 2]. Deficiency in safety learning could be a critical element of anxiety-related disorders [3, 4]. To translate safety learning into clinical research, a fundamental understanding of the process is vital. Pavlovian conditioned inhibition is considered a well-established experimental model for investigating safety learning. Safety learning can manifest in various ways, but it is best exemplified through differential conditioning. Different from extinction, which involves repeated exposure to conditioned fear-provoking stimuli until fear responses show a significant decrease [5–8], safety learning associates safety cues with the non-occurrence of aversive events [3, 9, 10]. Following repeated presentations, the stimulus that predicts the absence of aversive stimulus develops safe properties that are capable of inhibiting threat responses referred to as conditioned inhibition [11, 12]. Safety learning helps alert organisms to safe environments, fostering rewarding behaviors, such as feeding and mating, while dangerous environments inhibit these behaviors. Studies have shown that safety signals increase lever-pressing behavior for food in rats, whereas danger cues suppress it [13]. Even in Drosophila, conditioned approach behavior is increased when safety is signaled [14].

The neural circuits of safety learning are less well understood than those of fear learning. Single-unit recordings in the basolateral amygdala (BLA) of rats have demonstrated a sub-population of neurons within the BLA exhibited a comparable alteration in spike rate when exposed to both the safety cue and the reward cue, suggesting the shared encoding mechanisms for safety and reward within the BLA [3]. The fear-inhibitory and rewarding attributes of a safety signal can overlap, ultimately influencing responses to threats. For example, we previously showed that safety cues elevate the activity of dopaminergic neurons in the ventral tegmental area (VTA), a process necessary for the manifestation of safety learning. Importantly, the VTA is widely recognized to be involved in rewarding behavior, suggesting the safety cue shares similarities with the reward cues [9]. Additionally, VTA dopaminergic neurons play a crucial role in the enhancement of PV-INs activity within the PFC through a learning process. This elevated activity of PV-INs plays a pivotal role in inhibiting freezing responses associated with the safety cues [9].

The paraventricular thalamus, positioned ventral to the dorsal third ventricle [15], has been regarded as an important node in the limbic system. Studies have found that PVT neurons show heightened activity to a conditioned fear tone [16], and microinjection of muscimol into the PVT resulted in decreased freezing to a conditioned stimulus 24 h after conditioning [17]. Exposure to stressful environmental encounters is a risk factor for the development of anxiety-related disorders [18, 19]. Previous research from our laboratory have shown that PVT neurons are activated in response to restraint stress [20–22]. These activated PVT neurons project to the SOM-INs within the prelimbic cortex (PL) which subsequently preferentially inhibit PV-Ins [20]. Furthermore, the reduced activity of PL PV-INs in turn disinhibits the activity of PL projection neurons (PNs) that control the formation and expression of conditioned fear [20, 23]. Thus, the PVT may modulate both the formation and expression of aversive memory by tuning inhibitory functions within the prefrontal circuitry. However, it is unknown whether PVT also plays a role in modulating safety learning. Here we have addressed this question with a combination of calcium imaging, optogenetic and chemogenetic manipulations of neuronal activity in the PVT and PFC. Our results indicate that PVT is involved in both the formation and expression of safety learning. This modulation occurs through the activation of SOM-INs in PL, which subsequently inhibit PV-INs. Additionally, the circuitry plays a role in mediating the effect of stress on safety learning modulation. These findings may contribute to our understanding of stress-coping strategies concerning safety learning and have implications for the potential treatment targets.

## Materials and Methods

### Animals

C57BL6/J mice were obtained from Guangdong Medical Laboratory Animal Center, China. PV-Cre mice (B6; 129P2-Pvalbtm1 (Cre) Arbr/J, Jackson Laboratory, stock No.008069) were kindly provided by Dr. Miao He (Fudan University). Male C57BL6/J, SOM-Cre and PV-Cre mice were socially housed (5-6 mice/cage) under a 12 h light/dark cycle (8:00 AM–20:00 PM), and they were provided with food and water *ad libitum*. Adult male and female mice of 2–4 months old were used. All animal experiments were conducted in accordance with ARRIVE (Animal Research: Reporting of In Vivo Experiments) guidelines, approved by the Peking University Shenzhen Graduate School.

### Behavioral testing

Mice were handled and habituated to the conditioning context A, which has a grid floor connected to a shock generator (Coulbourn Instruments). The test context B was located inside a sound-attenuated cabinet (Coulbourn Instruments). Context A floors were cleaned with 1% acetic acid, while context B with 75% ethanol before retrieval experiments. During conditioning, context A was illuminated, and behavior was captured with a monochrome CCD camera and stored on a personal computer. In the strong foot-shock (0.75 mA) fear conditioning paradigm, on day 0, mice went through a habituation session in context B, in which they received 4 presentations of CS+ and CS- (30 s, 80 dB; CS+: 50 ms pips tone 3 kHz; CS-: white noise). Strong evidence suggests that latent inhibition may not occur with a pre-exposure to CS less than 17 times [24, 25]. In our study, the habituation consists of 4 CS− and 4 CS+, which is fewer than 17. Thus, this protocol is unlikely to induce significant latent inhibition. The inclusion of a habituation step aims to eliminate any potential startle response resulting from exposure to unfamiliar or un-encountered sounds, and this procedure has been used in several elegant studies [9, 26–28]. On day 1, mice received 3 CS+ paired with the US (2 s foot shock, 0.75 mA, inter-trial interval (ITI): 90 s). On day 2, conditioned mice were tested for retrieval in context B with 4 presentations of CS+ and CS−. Safety learning was conducted on day 3, which consists of 6 presentations of CS+ and CS- in a pseudorandom manner (ITI: 90 s) in context A, with CS+ paired with foot shock while CS- never reinforced with foot shock. On day 4, mice were tested for safety learning retrieval in context B with 4 presentations of CS+ and CS−. The discrimination index (DI) was computed from freezing levels to CS+ and CS− with the following formula: DI = ([CS+− CS−]/ [CS++CS−]). In our experiments, we utilized a strong foot-shock (0.75 mA) fear conditioning paradigm to examine both the fear conditioning and safety learning processes in the same mouse. It is noteworthy that, in the experiments aimed at investigating the effects of neuronal or circuitry manipulation on safety memory expression, we conducted safety learning retrieval for three consecutive days following the safety learning session.

In the weak foot-shock (0.3 mA) fear conditioning paradigm, on day 0, mice went through a habituation session in context B, in which they received 4 presentations of CS+ and CS− (30 s, 80 dB; CS+: 50 ms pips tone 3 kHz; CS−: white noise). On day 1, mice received 3 CS+−US pairing (2 s foot shock, 0.3 mA, and inter-trial interval: 90 s). On day 2, conditioned mice were tested for retrieval in context B with 4 presentations of CS+ and CS−.

To synchronize behavioral protocols and Ca^2+^imaging, transistor-transistor logic (TTL) pulses were generated from FreezeFrame (Coulbourn Instruments), and the TTL signals were recorded by the Fiber Recording System (Nanjing Thinker Tech). To synchronize behavioral protocols and optogenetic stimulation, TTL pulses were generated from FreezeFrame (Coulbourn Instruments) and the TTL signals were recorded by Optogenetic System (Inper LLC).

### Virus injection

Mice were anesthetized with isoflurane (5%) in an induction chamber and positioned in a stereotaxic frame anesthetized with isoflurane (2.5%) through a facemask. For virus injection experiments, a 5 μl syringe (Hamilton) was used to infuse at a rate of 60 nL/min. Viral constructs were injected bilaterally into the PVT (350 nL) and into the PL (300 nL). BrainVTA (Wuhan, China) was the source of all viruses used. Table 1 contains details regarding the virus. After the infusion, the needle was kept in place for an extra 5 min and removed slowly at a speed of 0.04 mm/s. A heating pad within a temperature range of 35–37 °C was used to maintain the body temperature of mice. Viral injection at the following stereotaxic coordinates: PVT (AP:−1.4 mm, ML: 0.07 mm DV: −3.2 mm), PL (AP: +2.1 mm, ML: ±0.3 mm, DV: −2.1 mm).

**Table 1:** virus used.

Bacterial and virus strains	Identifier	Virus titer	Source
AAV- CaMKIIa-GCaMP6s	Cat# PT-0110	≥2.00E+12vg/mL	BrainVTA
rAAV-CaMKIIa-hM3D(Gq)-EGFP-WPRE	Cat# PT-0525	≥2.00E+12vg/mL	BrainVTA
rAAV-EF1α-DIO-hM3D(Gq)-EYFP-WPRE-hGH	Cat#PT-0816	4.68E + 12 vg/mL	BrainVTA
rAAV-EF1α-DIO-EYFP-WPRE-hGH	Cat# PT-0012	5.24E + 12 vg/mL	BrainVTA
AAV-CaMKIIa-EYFP-WPRE	Cat# PT-0107	2.75E+12vg/ml	BrainVTA
rAAV-hSyn-Cre-WPRE-hGH	Cat# PT-0136	5.27E + 12 vg/mL	BrainVTA
rAAV-Ef1α-DIO-hM3D(Gq)-mCherry-WPREs	Cat# PT-0042	5.54E + 12 vg/mL	BrainVTA
rAAV-CaMKIIa-hM4D(Gi)-mCherry-WPRE	Cat# PT-0017	5.33E + 12 vg/mL	BrainVTA
rAAV-cfos-tTA-WPRE-hGH	Cat# PT −3022	2.71E + 12 vg/mL	BrainVTA
rAAV-Tre3g-hChR2-mcherry-WPRE	Cat#PT-0515	5.68E + 12 vg/mL	BrainVTA
rAAV-TRE3g-NpHR3.0-EYFP-WPRE-hGH	Cat#PT-0760	5.05E + 12 vg/mL	BrainVTA
rAAV-EF1α-DIO-GCaMp6s-WPRE-hGH	Cat# PT-0071	≥2.00E+12vg/mL	BrainVTA
rAAV-EF1α-DIO-hChR2(H134R)-EYFP-WPRE	Cat#PT-0001	2.07E+12vg/mL	BrainVTA
rAAV-CaMKIIa-hM3D(Gq)-mCherry-WPREs	Cat# PT-0020	4.54E + 12 vg/mL	BrainVTA
rAAV-EF1α-DIO-hM4D(Gi)-mCherry-WPREs	Cat#PT-0043	2.00E+12vg/mL	BrainVTA
rAAV-EF1a-DIO-mCherry-WPRE	Cat#PT-0013	5.76E + 12 vg/mL	BrainVTA
rAAV-CaMKIIa-mcherry-WPRE	Cat#PT-0108	2.07E+12vg/mL	BrainVTA
rAAV-CaMKIIa-hChR2-mCherry-WPRE-hGH	Cat#PT-0005	5.75E + 12 vg/mL	BrainVTA

### Fiber photometry and data analysis

Mice were habituated to handling (5 min) and patch cord tethering (10 min) for 3 consecutive days before the start of the imaging experiments. The Ca^2+^ fluorescence signals were recorded throughout the entire behavior experiments (habituation, fear conditioning, retrieval (post-FC), safety learning, retrieval (post-SL). To record Ca^2+^ responses in PVT neurons, mice were injected with the AAV2/9- CaMKIIa-GCaMP6s virus in PVT. To record Ca^2+^ responses in PVT stress neurons, mice were injected with the AAV-cFos-tTA-pA and AAV-TRE3g-jGCaMP6s virus in PVT. To record Ca^2+^ responses in SOM-INs, SOM-Cre mice were injected with the AAV2/9-Ef1α-DIO- GCaMP6s virus in the PL. To confirm that stress-activated PVT neurons affect the expression of safety learning, we adopted a strategy to selectively express Ca^2+^ indicator GCaMP6s in stress-activated PVT neurons using the c-Fos/tTA system. This method links the promoter of c-Fos, an immediate early gene, with the tetracycline transactivator (TTA), a crucial element of the doxycycline (Dox) system that enables controllable expression of a specific gene. When dox is present, the binding of c-fos promoter-driven-TTA to its intended tetracycline-responsive element (TRE) site is impeded, consequently hampering the activation of GCaMP expression. However, when Dox is absent, acute restraint stress results in the selective labeling of GCaMP protein in the active neurons [29].

Following virus injection, ceramic ferrules (Inper LLC) were implanted and directed toward target brain regions. Ferrules were fixed in place using dental cement. Fluorescence signals of GCaMP were acquired and recorded using a fiber photometry system (Nanjing Thinker Tech, China). Blue light (470 nm LED) was transmitted into the brain at 20–30 µW and kept constant across experimental sessions. The emitted light was band-pass filtered (MF525-39, Thorlabs) and collected using CMOS (DCC3240M, Thorlabs) with a sampling rate of 50 frames per second. Fiber photometry data were analyzed using MATLAB. Changes in fluorescence (ΔF/F) during behavior were calculated as (F-F_0_)/F_0_, where F_0_ is the averaged baseline fluorescence within 1.5 s (between −2 s to −0.5 s before the event). ΔF/F values are presented as heat maps or averaged value plots. The area under the curve was calculated for a period of 500 ms before and 500 ms after the peak of fluorescence responses.

### In vivo pharmacogenetic manipulations

Mice were injected with the AAV2/9-CaMKIIa-hM4D (Gi) virus in the PVT to inhibit PVT excitatory neurons. To activate PVT excitatory neurons, mice were injected with the AAV2/9-CaMKIIa-hM3D (Gq) virus. To activate PL projecting PVT neurons, mice were injected with the AAV2/retro-hSyn-Cre virus in PL and the AAV2/9-Ef1α-DIO-hM3D (Gq) virus in PVT. Four weeks later, mice were injected intraperitoneally with CNO (3.3 mg/kg, MedChemExpress) 30 min before either safety learning or safety memory retrieval.

### In vivo optogenetic manipulations

To inhibit PVT stress neurons, we injected AAV-cFos-tTA-pA and AAV-TRE3g-NpHR3.0 virus in PVT with opto-fiber implanted in PVT. To activate stress activated neurons in PVT, we injected AAV-cFos-tTA-pA and AAV-TRE3g-hChR2 (E123T/T159C) in PVT with optic fiber implanted in PVT. To activate PL SOM-INs, SOM-Cre mice were injected with AAV2/9-Ef1α-DIO-ChR2 virus in PL, with opto-fiber implanted in PL. For trials including optogenetic stimulation, a final transmitted intensity of 10–15 mW for 473 nm (for ChR2; 20 Hz, 20 ms) or 593 nm (for NpHR; constant light) was used. Optogenetic manipulations in the behavioral tasks were performed with a controlled design, with alternating light-off and light-on. The blue light or yellow light was switched on at the CS onset and was switched off immediately following CS termination (30 s total duration).

### Histology

Histological procedures were performed on mice in this study. An overdose of sodium pentobarbital was used to anesthetize the mice, after which they were perfused through the heart with 0.9% PBS followed by a fixative solution containing 4% paraformaldehyde in PBS. The brains were then removed and post-fixed in the aforementioned fixative solution at 4 °C overnight. The samples were subsequently transferred to a sucrose solution consisting of 30% sucrose in PBS and stored at 4 °C for 48 h. Coronal sections with a thickness of 40 μm were analyzed to confirm the expression of the virus in the brain and ensure that the optical fiber had been properly placed.

### Data analysis

The choice of sample size reflects the nature of the experiment and statistical considerations. In this study, the majority of our data involved within-subject comparisons, where the same mouse was assessed before and after the experimental manipulations. This experimental design allowed us to minimize individual variations and hence reduced the number of animals needed to achieve statistical significance. Emphasizing our commitment to the reduction principle and the ethical considerations associated with animal research, we believe that the chosen sample size achieves an appropriate balance between scientific vigor and minimizing animal use. Statistical significance was calculated using two-tailed paired/unpaired t-test, one-way/two-way RM ANOVA (Version 8.0, GraphPad Prism software, USA), as noted. Data are reported as mean ± SEM. Significance levels are noted as *P < 0.05, **P < 0.01, ***P < 0.001, ****P < 0.0001.

## Results

### Safety learning modulates CS-induced PVT neuron responses

**Fig. 1:**
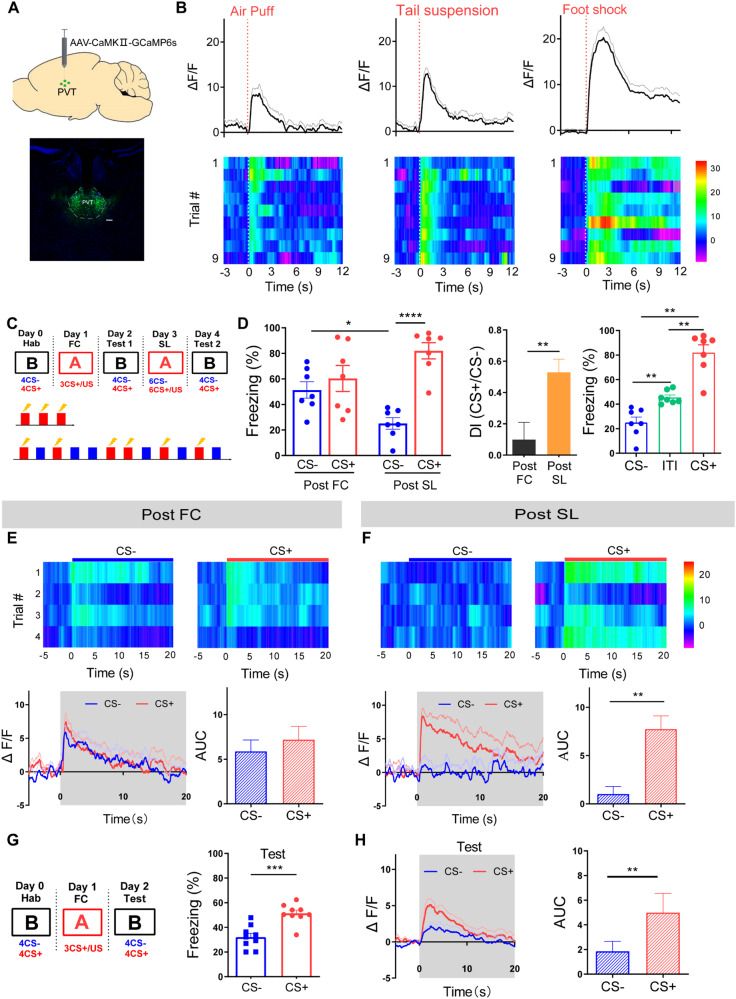
Differential responses of PVT excitatory neurons to CS- and CS+ after fear condition and safety learning. **A** Injection site of GCaMP6s virus for monitoring Ca^2+^ transients in PVT neurons. Scale bar: 100 μm. **B** Average and heat map of Ca^2+^ transients in response to different stimuli (air puff, tail suspension, and foot shock). **C** Experimental protocol for fear conditioning and safety learning. **D** Freezing levels(Left) during CS- and CS+ retrieval and discrimination index (middle) 24 h after FC and safety learning (two-way ANOVA, interaction, F(1, 24) = 11.21, *P* < 0.01, stimulus, F(1, 24) = 21.20, *P* < 0.0001, training, F(1, 24) = 0.09946, *P* = 0.7552; *N* = 7 mice, CS-(post-FC)vs. CS+(post FC), *P* = 0.7663, CS-(post SL) vs. CS + (post SL), *P* < 0.0001, CS-(post FC) vs. CS-(post SL), *P* < 0.05, CS + (post FC) vs. CS + (post SL), *P* = 0.0846, Bonferroni’s post-test; two-tailed paired t-test, *t* = 4.191, df = 6; *N* = 7 mice, Post FC vs. Post SL, *P* < 0.01). Right: Freezing levels during CS-, inter-trial-interval (ITI) and CS + 24 h after SL (one-way ANOVA, *N* = 7 mice, CS- vs. ITI, *P* < 0.01, ITI vs. CS+, *P* < 0.01, CS- vs. CS+, *P* < 0.01, Bonferroni’s post-test). **E** Heat maps of (upper) and averaged (lower) PVT neuron responses during CS- and CS+ post-fear conditioning (two-tailed paired t-test, *t* = 1.509, df = 6; *N* = 7 mice, CS- vs. CS+, *P* = 0.182). **F** Heat maps of (upper) and averaged (lower) PVT neuron responses during CS- and CS+ after safety learning (two-tailed paired t-test, *t* = 3.823, df = 6; *N* = 7 mice, CS- vs. CS+, *P* < 0.01). **G** The weak foot shock (0.3 mA) fear conditioning protocol and freezing levels during CS- and CS+ retrieval 24 h after FC (two-tailed paired t-test, *t* = 4.734, df=16; *N* = 9 mice, CS- vs. CS+, *P* < 0.01). **H** Averaged PVT neuron responses during CS- and CS+ post-FC (two-tailed paired t-test, *t* = 3.534, df =8; *N* = 9 mice, CS- vs. CS+, *P* < 0.01).

To probe the sensitivity of PVT neurons to threatening events, we randomly labeled PVT excitatory neurons using the AAV-CaMKIIa-GCaMP6s virus. The expression of the GCaMP6 virus allows monitoring of neuronal activity by measuring changes in the fluorescence intensity collected via an optic fiber imbedded in the PVT (Fig. 1A). We found significant Ca^2+^ responses to various acute stressors, including air puff, tail suspension and foot shock (Fig. 1B). These findings are consistent with previous research indicating that PVT is activated by a variety of stressors [30–34]. In our previous study, we established an experimental paradigm facilitating the transition from generalized to discriminative fear responses in the same mouse via safety learning [9]. In the current study, we employed the same protocol to investigate the contribution of PVT to safety learning. The basic steps of the experimental paradigm are as follows: a fear generalization state will be induced with three pairings of CS+ and a high-intensity foot shock (US, 0.75 mA), followed by a safety learning process consisting of 6 presentations of CS+ and CS- in a pseudorandom manner. During safety learning, CS+ was paired with a foot shock, while CS- was not reinforced with a foot shock (Fig. 1C). To explore the role of PVT in safety learning, we measured freezing levels and PVT neuron activity after fear conditioning and safety learning (Fig. 1C [9]). After fear conditioning (FC), freezing levels were high during the presentation of both CS+ and CS-, with no significant difference between them, indicating a generalized fear state (Fig. 1D; [9]). After safety learning (SL), freezing levels during CS- were significantly lower than those during CS+ and the discrimination index was larger after SL, indicating a discrimination state (Fig. 1D [9]). It is noteworthy that, after safety learning, freezing levels during CS- were lower than those observed during the ITI (Fig.1D), indicating that this paradigm effectively conveys safety learning. Safety learning may involve the modulation of both contextual and cued fear. To investigate the potential contribution of contextual fear modulation, we examined the baseline freezing levels during the 3-minute exposure period to context B, 24 h after safety learning. We observed that the freezing levels were comparable to those observed during the post-FC period (Fig. S1). This suggests that context had a minimal effect on fear modulation following safety learning.

Simultaneously with behavioral tests, we measured Ca^2+^ responses in PVT neurons after fear conditioning and safety learning. After the fear conditioning, Ca^2+^ responses in PVT neurons were comparable in response to both CS+ and CS-, indicating that PVT neurons do not discriminate between CS+ and CS- at this generalization state (Fig. 1E). In contrast, after safety learning, PVT neuron responses to CS- were significantly reduced and smaller than the responses to CS+ (Fig. 1F). These results indicate that safety learning modulates CS- induced PVT neuronal responses.

To further investigate the impact of stimulus intensity on fear memory generalization and discrimination, and PVT neuron responses, we employed a weaker conditioning protocol. Notably, we observed differential freezing levels and PVT responses to the CS- and CS+ (Fig. 1G, H), indicating that the generalization/discrimination fear state and PVT neuron response are affected by the US intensity. Consequently, for the remainder of this study, we opted to use a strong conditioning protocol to investigate the specific contribution of PVT to safety learning.

### PVT neurons modulate the expression of safety learning

**Fig. 2:**
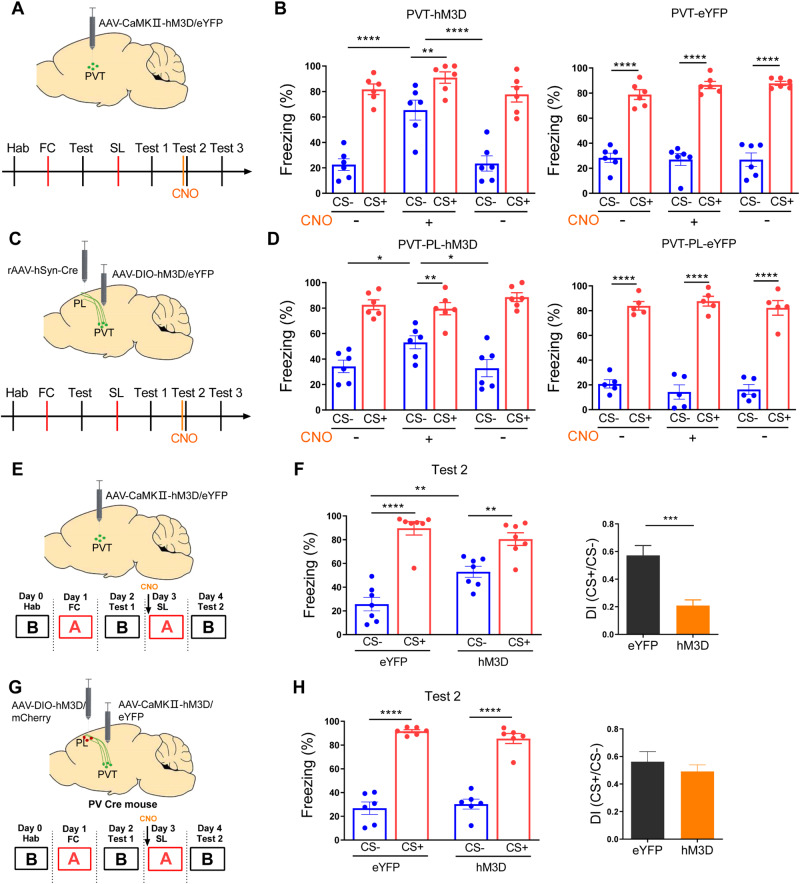
Activation of paraventricular thalamus (PVT) neurons impairs safety learning. **A** Schematic illustration of chemogenetic activation of PVT neurons. **B** Freezing levels during CS+ and CS− before, during, and after CNO injection in PVT-hM3D (Gq)- or eYFP-injected mice post-safety learning (two-way ANOVA, interaction, F(2,15) = 7.056, *P* < 0.01, stimulus, F(1,15) = 138.1, *P* < 0.0001 treatment, F(2,15) = 11.74, *P* < 0.001; *N* = 6 mice, CS-(pre CNO) vs. CS-(CNO 30 min), *P* < 0.0001, CS-(CNO 30 min) vs. CS-(post CNO), *P* < 0.0001, Bonferroni’s post-test; two-way ANOVA, interaction, F(2,15) = 0.9325, *P* = 0.4152, stimulus, F(1,15) = 281.0, *P* < 0.0001, treatment, F(2,15) = 0.6340, *P* = 0.5441; *N* = 6 mice, CS-(pre CNO) vs. CS-(CNO 30 min), *P* > 0.99, CS-(CNO 30 min) vs. CS-(post CNO), *P* > 0.99, Bonferroni’s post-test). **C** Schematic illustration of chemogenetic activation of PL-projecting PVT neurons. **D** Freezing levels during CS+ and CS− before, during, and after CNO injection in PVT-PL hM3D (Gq)- or eYFP-injected mice post-safety learning(two-way ANOVA, interaction, F(2,15) = 4.846, *P* < 0.05, stimulus, F(1,15) = 118.1, *P* < 0.0001, treatment, F(2,15) = 1.363, *P* = 0.2859; *N* = 6 mice, CS-(pre CNO)vs. CS-(CNO 30 min), *P* < 0.05, CS-(CNO 30 min) vs. (post CNO), *P* < 0.05, Bonferroni’s post-test; two-way ANOVA, interaction, F(2,12) = 1.277, *P* = 0.3143, stimulus,F(1,12) = 610.6, *P* < 0.0001, treatment, F(2,12) = 0.1554, *P* = 0.8578; *N* = 5 mice, CS-(pre CNO)vs. CS-(CNO 30 min), *P* > 0.99, CS-(CNO 30 min) vs. CS-(post CNO), *P* > 0.99, Bonferroni’s post-test). **E** Procedure for chemogenetic activation of PVT neurons during safety learning. **F** Freezing levels (left) and discrimination index (right) post-SL in mice injected with hM3D virus or control virus(two-way ANOVA, interaction, F(1,24) = 11.59, *P* < 0.01, stimulus, F(1,24) = 73.43, *P* < 0.0001, treatment, F(1,24) = 2.880, *P* = 0.1026; eYFP, *N* = 7 mice, hM3D, *N* = 7 mice, CS-(eYFP)vs. CS-(hM3D), *P* < 0.01, CS + (eYFP) vs. CS + (hM3D), *P* = 0.4212, Bonferroni’s post-test; two-tailed unpaired *t* test, *t* = 4.443, df = 12; eYFP, *N* = 7 mice, hM3D, *N* = 7 mice, eYFP vs. hM3D, *P* < 0.001). **G** Procedure for chemogenetic activation of PVT neurons and activation of PL PV-INs during safety learning. **H** Freezing levels (left) and discrimination index (right) post-safety learning in mice injected with hM3D virus or control virus (two-way ANOVA, interaction, F(1, 20) = 1.249, *P* = 0.2769, stimulus, F(1, 20) = 216.8, *P* < 0.0001, treatment, F(1, 20) = 0.1666, *P* = 0.6857; Control, *N* = 6 mice, hM3D, *N* = 6 mice, CS-(Control) vs. CS-(hM3D), *P* = 0.8566, CS+(Control) vs. CS+(hM3D), *P* = 0.5008, Bonferroni’s post-test; two-tailed unpaired t test, *t* = 0.7256, df = 10; Control, *N* = 6 mice, hM3D, *N* = 6 mice, Control vs. hM3D, *P* = 0.4847).

To better understand the contribution of PVT to safety learning, we first examined whether PVT activity influences the expression of safety learning. We have previously observed that freezing levels remained relatively stable after multiple retrieval tests [9]. To mitigate individual variations, we used the established protocols in which the same mouse was assessed before and after the experimental manipulations. To activate PVT neurons, we injected the AAV-CaMKIIa-hM3D virus into the PVT (Fig. 2A). The injection of CNO before the retrieval test resulted in a significant increase in freezing levels to CS- compared to the pre-CNO period. However, this effect dissipated after 24 h (Fig. 2B). In control experiments, no effect of CNO was seen (Fig. 2B). These results indicate the activation of PVT by CNO treatment resulted in an increase in freezing to CS-, while CNO treatment itself did not lead to changes in behavior. Furthermore, it’s important to note that PVT stimulation does not increase freezing in unconditioned mice (Fig. S2).

To examine whether PVT may affect safety learning in a sex-dependent manner, we repeated the above experiments in a cohort of female mice. Women are more likely than men to experience post-traumatic stress disorder [35], and hence there might be a sex-dependent difference in safety learning. When CNO was injected prior to the retrieval test, freezing levels were much higher than they were either before or after the CNO injection (Fig. S3). These findings suggest a similar contribution of PVT to the expression of formed safety memory in male and female mice.

Previous studies have provided evidence that the PVT projects to the PL [36, 37], and these projections are required to mediate stress impact on the expression of probabilistic fear conditioning [20]. To determine the impact of these projections on the expression of safety memory, we injected AAV-DIO-hM3D virus into the PVT and AAV-Retro-Cre virus into the PL to selectively activate PVT-to-PL projections (Fig. 2C). The injection of CNO before retrieval test led to a significant increase in freezing levels compared to those before or after CNO injection (Fig. 2D). No effect on freezing levels was observed after CNO injection in mice injected with the control eYFP virus (Fig. 2D). These results indicate that PVT-to-PL projections reversibly modulate the expression of safety learning.

### PVT neurons modulate safety learning in a PL PV-INs-dependent manner

To examine whether PVT activity also affects the learning of safety signals, we injected the AAV-CaMKIIa-hM3D/eYFP virus into the PVT (Fig. 2E). The injection of CNO before safety learning resulted in significantly higher freezing levels to CS- and lower discrimination index compared to mice injected with eYFP/control virus in retrieval test (Fig. 2E, F). This result indicates that safety learning is inhibited by PVT activation. Conversely, we injected AAV-CaMKIIa-hM4D or eYFP virus into the PVT to selectively inhibit PVT neurons. On the day of safety learning, CNO was injected to inhibit PVT neurons. Twenty-four hours after safety learning, CS-induced freezing levels were comparable to those of control mice, indicating that PVT inhibition did not affect safety memory formation. (Fig. S4). The activation of PL PV-INs is necessary for safety learning [9], and PVT activates PL SOM-INs which inhibit PV-INs in probabilistic fear learning [20]. It is possible that the activation of PVT may suppress PV-INs and impair safety learning. To test this hypothesis, we injected the AAV-CaMKIIa-hM3D virus into the PVT and AAV-DIO-hM3D virus into the PL of PV-Cre mice to simultaneously activate PVT neurons and PL PV-INs (Fig. 2G). Mice injected with hM3D or eYFP/control virus were given a CNO injection 30 min before safety learning (Fig. 2G). On the retrieval day, no significant differences in freezing levels or discrimination index were found between these two groups of mice (Fig. 2H). Together, these results indicate that PVT-induced impairment in safety learning likely occurs via the suppression of PL PV-INs activation.

### Stress-activated PVT neurons mediate safety learning modulation

**Fig. 3:**
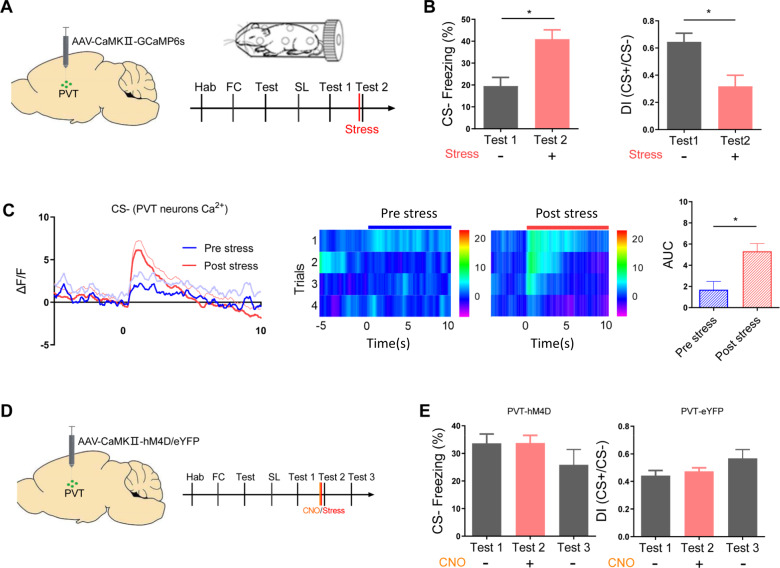
PVT mediates stress effects on safety learning modulation. **A** Schematic of GCaMP6 virus injection in PVT (left), and procedure for the stress impacts on PVT neuron activity and safety learning expression. **B** Freezing levels during CS− (left) and discrimination index (right) before and after acute stress in PVT-GCaMP6-injected mice (two-tailed paired t-test, *t* = 3.087, df = 6; *P* < 0.05; CS-(Test 1) vs. CS-(Test2); *N* = 7 mice; two-tailed paired t-test, *t* = 2.951, df = 6; *P* < 0.05; CS- (Test 1) vs. CS- (Test2); *N* = 7 mice). **C** Averaged (left) Ca^2+^ responses in PVT neurons during CS− before and after acute stress and its heat maps (middle). (Right) Mean Ca^2+^ responses (AUC) during CS- before and after stress (two-tailed paired t-test, t = 3.479, df = 6; *P* < 0.05; CS-(Test 1) vs. CS-(Test2); *N* = 7 mice). **D** Schematic of acute stress exposure and chemogenetic inhibition of PVT neurons after safety learning. **E** Freezing levels (left) and discrimination index (right) during CS− before, during and after acute stress exposure with PVT chemogenetic inhibition (one-way ANOVA, F(2,15) = 1.225, *P* = 0.3215; *N* = 6 mice, test 1(pre-stress/ CNO) vs. test 2 (stress/ CNO), *P* > 0.99, test 2(stress/CNO)vs. test 3(post stress/CNO), *P* = 0.5743, Bonferroni’s post-test; one-way ANOVA, F(2,15) = 2.099, *P* = 0.1572; N = 6 mice, test 1(pre stress/CNO)vs. test 2(stress/CNO), *P* > 0.99, test 2 (stress/CNO)vs. test 3(Post stress/CNO), *P* = 0.4802, Bonferroni’s post-test).

By implementing a well-established and relatively severe stress protocol [38–40], our previous studies have demonstrated that PVT plays a pivotal role in mediating the impact of acute stress on the expression and formation of probabilistic fear memory [20, 23]. In the current study, we used the same stress protocol to gain insights into the mechanisms and circuitry mediating the impact of acute stress on safety memory. To examine whether stress-activated PVT neurons also mediate the impaired expression of safety learning, we first monitored PVT Ca^2+^ activity by injecting the AAV-CaMKIIa-GCaMP6s virus into the PVT (Fig. 3A). Our findings revealed that 2-hour restraint stress led to higher freezing levels and lower discrimination index compared to the pre-stress condition (Fig. 3B). In addition, the acute stress led to larger CS-induced Ca^2+^ responses in PVT neurons (Fig. 3C). To examine whether the elevated PVT neuron activity is required for stress-induced impairment in the expression of safety learning, we injected AAV-CaMKIIa-hM4D virus into the PVT to inhibit the activity of PVT excitatory neurons. Before subjecting the mice to stress, a CNO injection was administered (Fig. 3D). In contrast to the results that acute stress impaired the expression of safety memory (Fig. 3A, B). This procedure abolished the changes in freezing and discrimination index induced by acute stress (Fig. 3E). These results indicate that PVT may mediate stress-induced impairment in the expression of safety learning.

**Fig. 4:**
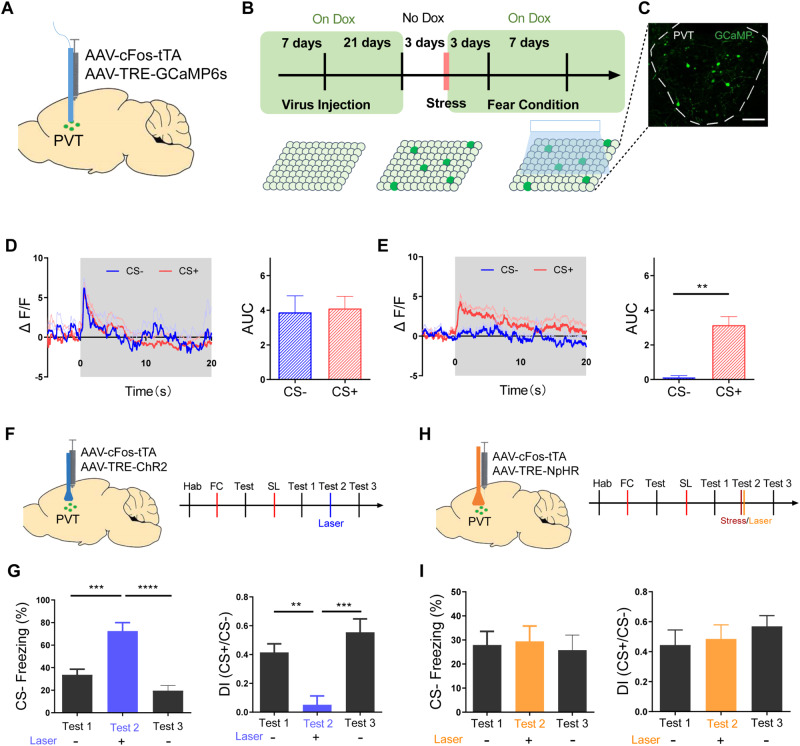
Stress-activated neurons in the PVT modulate safety learning. **A** Mice injected with c-Fos-tTA and TRE-GCaMP6 virus, with an optical fiber implemented into the PVT. **B** Experimental procedure for labeling stress-response neurons in the PVT. **C** Representative image showing the expression of GCaMP6 in stress-activated neurons in PVT. Scale bar: 100 μm. **D** Averaged Ca^2+^ responses in the PVT stress-activated neurons during CS− and CS+ after fear conditioning (two-tailed paired t-test, *t* = 0.3114, df = 5; *N* = 6 mice, CS- vs. CS+, *P* = 0.7681). **E** Averaged Ca^2+^ responses in the PVT stress neurons during CS− and CS+ after safety learning (two-tailed paired t-test, *t* = 6.542, df = 5; *N* = 6 mice, CS- vs. CS+, *P* < 0.01). **F** Schematics for injections of c-Fos-tTA and TRE-ChR2 virus in the PVT (left) and experimental procedure (right). **G** Freezing levels (left) and discrimination index (right) during CS- retrieval before, during, and after opto-stimulation of PVT stress-activated neurons (one-way ANOVA, F(2,10) = 37.32, *P* < 0.0001; *N* = 6 mice, test 1(laser off)vs. test 2 (laser on), *P* < 0.001, test 2 (laser on)vs. test 3 (laser off), *P* < 0.0001, Bonferroni’s post-test; one-way ANOVA, F(2,10) = 21.13, P < 0.001; *N* = 6 mice, test 1(laser off)vs. test 2(laser on), *P* < 0.01, test 2 (laser on)vs. test 3 (laser off), *P* < 0.001, Bonferroni’s post-test). **H** Schematics for injections of c-Fos-tTA and TRE-NpHR virus in the PVT (left) and experimental procedure (right). **I** Freezing levels (left) and discrimination index (right) during CS- retrieval before, during and after opto-stimulation of PVT stress-activated neurons (one-way ANOVA, F(2,12) = 0.1201, *P* = 0.8879; *N* = 7 mice, test 1 (laser off) vs. test 2 (laser on), *P* > 0.99, test 2 (laser on) vs. test 3 (laser off), *P* > 0.99, Bonferroni’s post-test; one-way ANOVA, F(2,12) = 0.7088, *P* = 0.5117; *N* = 7 mice, test 1 (laser off) vs. test 2 (laser on), *P* > 0.99, test 2 (laser on) vs. test 3 (laser off), *P* > 0.99, Bonferroni’s post-test).

In the experiments described previously, PVT neurons were randomly labeled or tagged, which introduces the possibility that the neurons being manipulated may not be those activated during stress. To address this concern, we employed a virus combination consisting of AAV-cFos-tTA and AAV-TRE-GCaMP6s, which was injected into the PVT (Fig. 4A). During the virus expression period, mice were fed on Doxycycline (Dox) as part of their diet. After virus expression, we subjected mice to acute restraint stress while off Dox, and then reinstated Dox for the behavioral test (Fig. 4B). This approach allowed us to achieve GCaMP expression in stress-activated neurons within the PVT (Figs. 4C and S5). Next, we investigated whether these labeled neurons affect safety learning by recording their activity with fiber photometry. During the fear generalization state, PVT stress-activated neurons were active during both CS+ and CS-, with comparable area under curve (AUC) values (Fig. 4D). In contrast, during the discrimination state, the responses to CS- were significantly smaller than those elicited by the CS+ stimulus (Fig. 4E). These results indicate that safety learning leads to reduced CS- responsiveness in stress-activated neurons in the PVT.

Next, we aimed to investigate whether manipulating these stress-activated PVT neurons could result in a bidirectional modulation of safety learning. To achieve this, we injected AAV-cFos-tTA and AAV-TRE-ChR2 viruses into the PVT (Fig. 4F). After safety learning, the activation of PVT stress-responding neurons led to significantly higher freezing levels during CS- than before or after this activation (Fig. 4G). This indicates that activation of PVT stress neurons can reversibly affect the expression of safety learning. Subsequently, we injected AAV-cFos-tTA and AAV-TRE-NpHR viruses into the PVT to inhibit the activity of PVT stress-activated neurons after subjecting the mice to stress (Fig. 4H). In contrast to the effects of acute stress on the expression of safety memory (Fig. 3A, B), optogenetic inhibition after stress abolished the impact of stress on freezing level and discrimination index (Fig. 4I). These results provide further evidence that PVT stress-activated neurons may mediate stress-induced impairments on the expression of safety learning.

### PVT regulates safety learning through modulation of PL SOM-INs

**Fig. 5:**
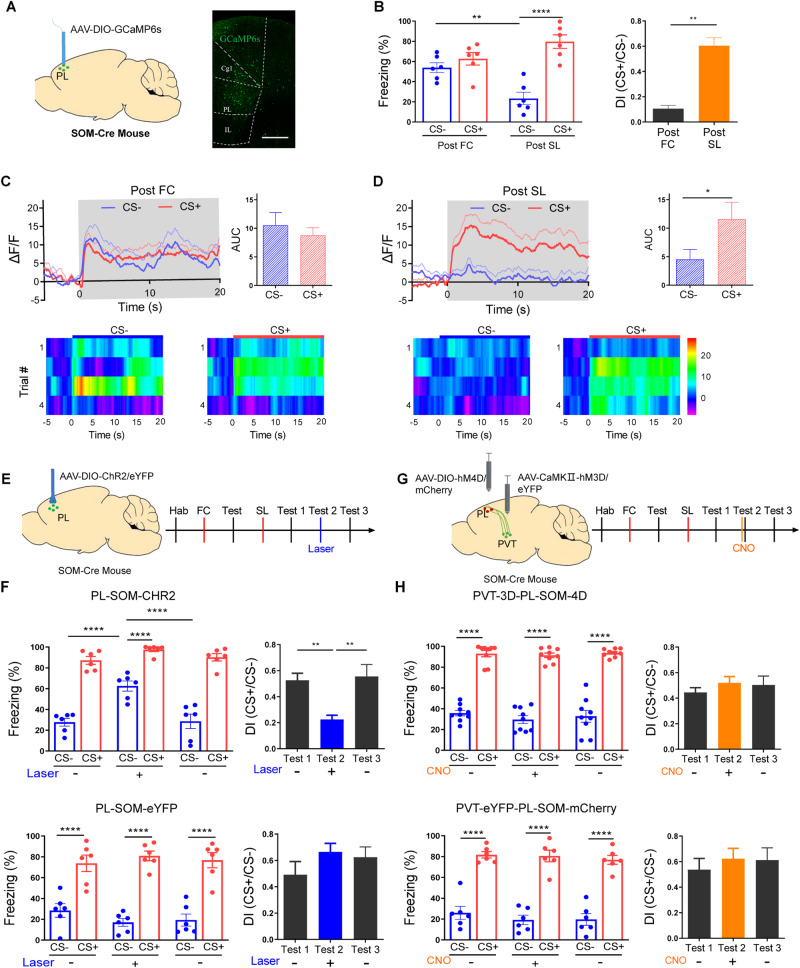
PVT regulates safety learning through modulation of PL SOM-INs. **A** Schematic of injection of GCaMP6 virus in PL of SOM-Cre mice and location of injection site. Scale bar: 500 μm. **B** Freezing during CS- and CS+ retrieval (left) and discrimination between CS- and CS+ (right) 24 h post-FC and post-SL (two-way ANOVA, interaction, F(1, 20) = 15.57, *P* < 0.001, stimulus, F(1,20) = 29.36, *P* < 0.0001, treatment, F(2,10) = 1.267, *P* = 0.2736; *N* = 6 mice, CS-(post FC)vs. CS-(post SL), *P* < 0.01, CS+ (post FC) vs. CS+ (post SL), *P* = 0.1163, CS-(post FC) vs. CS+ (post FC), *P* = 0.5242, CS-(post SL) vs. CS+ (post SL), *P* < 0.0001, Bonferroni’s post-test; two-tailed paired t-test, *t* = 6.224, df = 5; *N* = 6 mice, Post FC vs. Post SL, *P* < 0.01). **C** Averaged Ca^2+^ responses (upper) and heat maps (lower) in SOM-INs during CS− and CS+ after FC (two-tailed paired t-test, *t* = 0.4700, df = 5; *N* = 6 mice, CS- vs. CS+, *P* = 0.6581). **D** Averaged Ca^2+^ responses (upper) and heat maps (lower) in SOM-INs during CS− and CS+ post SL (two-tailed paired t-test, *t* = 3.043, df = 5; *N* = 6 mice, CS- vs. CS+, *P* < 0.05). **E** Schematics of injections of ChR2 or eYFP control virus in PL in SOM-Cre mice (left) and experimental procedure (right). **F** Freezing levels (left, PL-SOM-CHR2: (two-way ANOVA, interaction, F(2,15) = 10.67, *P* < 0.01, stimulus, F(1,15) = 399.0, *P* < 0.0001, treatment, F(2,15) = 10.97, *P* < 0.01; *N* = 6 mice, CS-(laser off)vs. CS-(laser on), *P* < 0.0001, CS-(laser on)vs.(laser off), *P* < 0.0001, Bonferroni’s post-test; PL-SOM-eYFP: two-way ANOVA, interaction, F(2,15) = 2.472, *P* = 0.1180, stimulus, F(1,15) = 257.8, *P* < 0.0001, treatment, F(2,15) = 0.08919, *P* = 0.9152; *N* = 6 mice, CS-(laser off) vs. CS-(laser on), *P* = 0.5883, CS-(laser on)vs.(laser off), *P* = 0.9913, Bonferroni’s post-test;) and discrimination index (PL-SOM-CHR2: one-way ANOVA, F(2,15) = 8.043, *P* < 0.01; *N* = 6 mice, test 1(laser off) vs. test 2(laser on), *P* < 0.01, test 2(laser on) vs. test 3(laser off), *P* < 0.01, Bonferroni’s post-test; PL-SOM-eYFP: one-way ANOVA, F(2,15) = 1.218, *P* = 0.3234; *N* = 6 mice, test 1(laser off)vs. test 2(laser on), *P* = 0.4702, test 2(laser on) vs. test 3(laser off), *P* > 0.99, Bonferroni’s post-test) during CS- and CS+ retrieval before, during, and after laser stimulation. **G** Schematics of injections of hM4D or mCherry control virus in PL and hM3D or eYFP control virus in PVT in SOM-Cre mice (left) and experimental procedure (right). **H** Freezing levels (left, PVT-3D-PL-SOM-4D: two-way ANOVA, interaction, F(2,24) = 0.2254, *P* = 0.7998, stimulus, F(1,24) = 410.3, *P* < 0.0001, treatment, F(2,24) = 0.7848, *P* = 0.4676; *N* = 9 mice, CS-(pre CNO) vs. CS-(CNO), *P* = 0.6217, CS-(CNO)vs.(post CNO), *P* > 0.99, Bonferroni’s post-test; PVT-eYFP-PL-SOM-mCherry: two-way ANOVA, interaction, F(2,15) = 0.1386,*P* = 0.8717, stimulus, F(1,15) = 167.8, *P* < 0.0001, treatment, F(2,15) = 0.8622, *P* = 0.4421; *N* = 6 mice, CS-(pre CNO)vs. CS-(CNO), *P* > 0.99, CS-(CNO) vs.(post CNO), *P* > 0.99, Bonferroni’s post-test) and discrimination index (right, PVT-hM3D-PL-SOM-hM4D: one-way ANOVA, F(2,24) = 0.5498, *P* = 0.5841; *N* = 9 mice, test 1(pre CNO)vs. test 2(CNO), *P* = 0.9826, test 2(CNO) vs. test 3(Post CNO), *P* > 0.99, Bonferroni’s post-test; PVT-eYFP-PL-SOM-mCherry: one-way ANOVA, F(2,15) = 0.2713, *P* = 0.7660; *N* = 6 mice, test 1(pre CNO)vs. test 2(CNO), *P* > 0.99, test 2(CNO)vs. test 3(post CNO), *P* > 0.99, Bonferroni’s post-test) during CS- and CS+ retrieval before, during, and after CNO injection.

Our previous studies have demonstrated that PVT inputs excite PL SOM-INs, which in turn inhibit PL PV-INs and disinhibit PL excitatory neurons, resulting in increased freezing levels [20]. To investigate whether a similar circuitry and mechanism are involved in the impact of stress on safety learning, we injected the AAV-DIO-GCaMP6s virus into the PL and the AAV-CaMKIIa-ChR2 virus into the PVT of SOM-Cre mice (Fig. S6A). This allowed us to observe that PVT optogenetic stimulation resulted in increased PL SOM-INs activity compared to mice expressing mCherry virus (Fig. S6B, C). We then injected the AAV-DIO-GCaMP6s virus into the PL of SOM-Cre mice and recorded SOM-INs activity following fear conditioning and safety learning (Fig. 5A). Following fear conditioning, we observed comparable responses to CS+ and CS- in SOM-INs, as well as similar freezing levels (Fig. 5B, C). However, following safety learning, the responses and freezing levels induced by CS+ were significantly larger than those induced by CS- (Fig. 5B, D). This reduction in SOM-INs responses following safety learning may reflect a diminished PVT input.

To investigate how SOM-INs may modulate safety learning, we manipulated their activity by injecting the AAV-DIO-ChR2 virus into the PL of SOM-Cre mice (Fig. 5E). In naïve mice, optical activation of SOM-INs elicited significant freezing (Fig. S7). Moreover, after safety learning, optogenetic activation of SOM-INs resulted in significantly higher freezing levels and lower discrimination index compared to the levels observed before and after SOM-INs activation. Notably, no such effects were observed in mice injected with the eYFP/control virus (Fig. 5F). These results indicate that PL SOM-INs modulate the expression of safety learning. We then tested whether the activation of PL SOM-INs is necessary for PVT-elicited freezing. In contrast to the results that where chemogetic activation of PVT (Fig. 2A, B) and activation of SOM-INs (Fig. 5E, F) impeded the expression of safety memory, concurrent chemogenetic activation of PVT and chemogenetic inhibition of PL SOM-INs negated the freezing-promoting effect of PVT activation (Fig. 5G, H). These findings imply that the activation of SOM-INs mediates PVT-induced impairment of safety learning. To examine whether this key modulation circuitry also functions in the female mice, we injected the AAV-DIO-hM3D virus into the PL of a cohort of female SOM-Cre mice, and observed similar effects as in male mice (Fig. S8). These results suggest that for the safety learning paradigm used in the current study, sex does not appear to play major role.

## Discussion

In this study, we showed that acute stress impairs the expression and formation of safety memories. Specifically, stress-activated PVT neurons excite PL SOM-INs, which subsequently suppress the activity of PL PV-INs. This inhibition ultimately leads to heightened fear responses to safety cues. These results demonstrate how acute stress affects safety learning by altering prefrontal inhibition. This highlights the intricate interplay among various brain regions and mechanisms involved in regulating adaptive responses to safety memory (Fig. S9).

### Stress modulation of safety memory formation and expression

The PVT has emerged as an important node within the limbic system, regulating both positive and negative behaviors [16, 41]. The PVT receives diverse cortical and subcortical inputs that participate in stress-associated processes [42–44]. Notably, the PVT is involved in the modulation of conditioned fear. For example, PFC projections to the PVT mediate the formation and maintenance of fear memories [45, 46]. In addition, PVT projects to the medial shell of the nucleus accumbens and modulates opiate-associated memories [47]. Inhibition of dense PVT projections to the central amygdala has been shown to impair fear memory retrieval [16]. Here, we observed a transient increase in PVT calcium activity following exposure to stressors such as air puff, tail suspension and foot shock stress exposure, which aligns with the previous reports of heightened activity of PVT neurons associated with stressors [30–34]. This activation is associated with higher basal activity of PVT neurons after stress exposure and enhanced responses to conditioned stimuli [20].

Our previous research have demonstrated that the PVT modulates the formation of probabilistic fear memory in a reversible and dynamic manner [20]. Our current findings are consistent with these earlier observations and further emphasize the critical contribution of PVT to conditioned inhibition shared by these two experimental paradigms. During safety learning, activation of PVT impairs the formation of safety memory. Subsequently, after safety learning, PVT activation reversibly modulates the expression of safety memory. Taken together, these findings suggest two key implications: (1) Elevated PVT activity can be seen as stress signal that temporarily suppresses the expression of conditioned inhibition, placing the animals in a heightened state of alertness and vigilance in the face of stress and potential danger. This heightened vigilance allows the animals to act in a more cautious manner. (2) High PVT activity during safety learning conditioning prevents the formation of safety memory, as stressed animals may be more inclined to perceive safety signals as potentially dangerous. This mechanism helps prevent the formation of false safety-related memories.

Accumulating evidence indicates that stress can affect the formation and expression of memory in a persistent [23, 48, 49] or dynamic way [23, 50, 51]. In both humans and rodents, emotional arousal promotes a bias towards the use of subcortical-dependent habit memory over frontal cortex-dependent flexible memory [52, 53]. The release of hormones and neurotransmitters released during stressful events is believed to modulate these memory systems [53]. In support of this idea, stress or cortisol administration has been shown to impair extinction retrieval, thus eliciting a return of fear [54–56]. Our previous study showed that PVT enables dynamic tuning of the probabilistic memory expression, thereby implicating a circuitry-based mechanism [20]. The findings from our current study indicate that a similar mechanism is involved in the PVT’s modulation of safety memory, influencing both the expression and formation of safety cue memory. As a further step, we have tagged, in an activity-dependent manner, the PVT neurons that are activated during acute stress and have demonstrated their contribution to the modulation of safety learning. Additionally, PVT inhibition blocks the impact of acute stress on the expression of safety learning. Together, these findings support the critical importance of PVT in mediating the impact of acute stress on the expression of safety memory.

One interesting finding that warrants future exploration is the observed training-induced alteration in PVT responses to CS. After fear conditioning and in the absence of discrimination between CS+ and CS-, PVT neurons respond similarly to CS+ and CS-. However, after safety learning with discrimination occurs, PVT neurons only respond to CS+. Understanding the mechanisms underlying the plasticity of PVT neurons in response to CS+, as well as what mediates the disappearance of CS- elicited responses in the same set of PVT neurons, presents an interesting avenue for future investigation.

### Contributions of SOM- and PV-INs to safety learning

In a recent study, we demonstrated that the interaction between PV- and SOM-INs plays an important role in the modulation of conditioned inhibition. Additionally, SOM-INs receive direct input from PVT projections, and this afferent evokes complex synaptic activity with a higher ratio of excitatory: inhibitory transmission in SOM-Ins [20]. This finding is consistent with SOM-INs and PV-INs exhibiting functionally complementary roles within the prelimbic circuitry [26, 57]. Our current results indicate that, following safety learning, the recruitment of SOM-INs decreases, which is associated with the freezing behavior elicited by reduced PVT afferent stimulation (Fig. S9). This indicates that even the modification of a sparse interneuron population can reorganize information processing in the local network. It has been confirmed that PV-INs are potent regulators of the synchronized activity and plasticity of PN [23, 58, 59]. SOM-INs activation may induce selective PV-INs inhibition and PN disinhibition [26], which may be due to PN receiving varying levels of inhibitory inputs from SOM-INs and PV-INs. The balance between the incoming activity from these GABAergic neurons could finally dictate how PN activity changes.

PV-INs provide powerful perisomatic inhibition to PN [58]. In a previous study, we have showed safety learning elevates the activity of PFC PV-INs [9]. It is plausible that the same population of PV-INs participating in the expression of safety cue-induced suppression of conditioned fear is inhibited by PVT inputs during stress exposure. This further supports the notion that PL PV-INs function as a hub to integrate diverse and distinct inputs [9, 23]. Additionally, this aligns with our recent finding that the same PL PV-INs mediate both safety cue and probabilistic cue-induced low fear responses [23]. The overarching model suggests that the subcortical circuitry enables a rapid but crude CS-US associations that only reflect their association at 100% with CS as a threat cue. In contrast, safety learning or low probability cues are mediated by PFC circuitry through a different conditioning/learning process and relies on inhibition in the PFC [9, 23]. Future studies will explore whether alterations in these PL PV-INs occur in disease states and the potential consequences of such changes.

### Other neuroanatomical regions involved in safety learning

In our study, we observed that activation of PVT impaired expression of safety memory, although there was still a significant difference between CS+/CS- after PVT activation. It is important to recognize that while our study primarily focused on the role of the PVT-PL circuit in the modulation of safety learning, this modulation can also be affected by other neural circuits. Recent research has implicated several neuroanatomical regions in safety learning and memory consolidation, including the amygdala, prefrontal cortex and VTA [11]. These regions may coordinate with the PVT to modulate safety learning. Neuroimaging studies have revealed a significant reduction in BOLD activity within the amygdala during exposure to safety cues, indicating decreased activation upon exposure to the safety cues [60]. In addition, studies conducted on rodents have showed a subpopulation of neurons in the BLA also exhibited similar responses to both safety and rewarding cues [3]. These findings suggest that the amygdala, known for its role in fear processing, may be involved in the modulation of safety learning.

In a previous study, we demonstrated that inputs from the ventral tegmental area (VTA) to PrL PV-INs are necessary for the formation of safety memory [9]. Specifically, plasticity in the VTA is required to support the elevated PL PV-INs activity during the expression of safety memory. Presentations of safety cues lead to enhanced activity in dopaminergic (DA) neurons [9]. Therefore, the relationship between the PVT-PL circuitry and the VTA-PL circuitry is of interest. We propose that these two circuits play distinct roles in the safety memory, but they converge onto PV-INs. Specifically, the VTA-PL-PV-INs pathway participates in the generation of the necessary plasticity along this pathway, leading to persistent changes in PV-INs activity associated with safety cues. In contrast, the PVT-PL-PV-INs pathway modulates the activity of PL-PV-INs in a reversal and non-plastic manner. Put another way, the VTA pathway determines the occurrence of plasticity of safety cue-associated changes in a persistent manner, while the PVT pathway determines dynamic expression rather than the memory itself. However, during memory formation, elevated activity of PVT neuron can veto this process by inhibiting PV-INs, and hence the absence of its activity is required. Thus, PVT-PL and VTA-PL circuits independently but coordinately modulate safety learning by converging onto PV-INs (Fig. S9).

## Conclusions

In summary, we demonstrate the impacts of stress on safety learning and elucidates a circuitry-based mechanism underlying this dynamic modulation. These findings provide valuable insights into the intricate processes underlying safety learning. The application of physical interventions in specific brain regions or neural circuits is a current hotspot for treating neurological diseases that are insensitive to medication. Such methods may include repetitive transcranial magnetic stimulation (rTMS) and deep brain stimulation (DBS). Considering that impaired safety learning is a prominent symptom in individuals with anxiety disorders, our research suggests that targeting PVT could potentially serve as a therapeutic target for anxiety treatment.

### Supplementary information

Supplementary Information for Modulation of learning safety signals by acute stress: paraventricular thalamus and prefrontal inhibition
