# Supplementary material for: Modulation of learning safety signals by acute stress: paraventricular thalamus and prefrontal inhibition
Source: Neuropsychopharmacology. 2024 Jan 5;49(6):961–73. doi: 10.1038/s41386-023-01790-2 (PMC11039638; doi:10.1038/s41386-023-01790-2)
Supplement: Supplementary file 1 — Supplementary Information for Modulation of learning safety signals by acute stress: paraventricular thalamus and prefrontal inhibition [file 41386_2023_1790_MOESM1_ESM.docx]

**Supplementary Information for**

**Modulation of learning safety signals by acute stress: paraventricular thalamus and prefrontal inhibition**

Zongliang Wang,^1^ Zeyi Wang,^1^ and Qiang Zhou^2^

Qiang Zhou Email: zhouqiang@pkusz.edu.cn

^1^State Key Laboratory of Chemical Oncogenomics, Guangdong Provincial Key Laboratory of Chemical Genomics, Peking University Shenzhen Graduate School, Shenzhen, PR China, 518055.

^2^State Key Laboratory of Chemical Oncogenomics, Guangdong Provincial Key Laboratory of Chemical Genomics, Peking University Shenzhen Graduate School, Shenzhen, PR China, 518055 zhouqiang@pkusz.edu.cn.

**This PDF file includes: Figs. S1-S9**

**Fig. S1. Baseline freezing levels during the 3-minute exposure period to the context B 24 hours after fear FC and SL.**

Baseline freezing levels during the 3-minute exposure period (two-tailed paired t test, t=0.1006, df = 6; N= 7 mice, CS- vs. CS+, P = 0.9232)


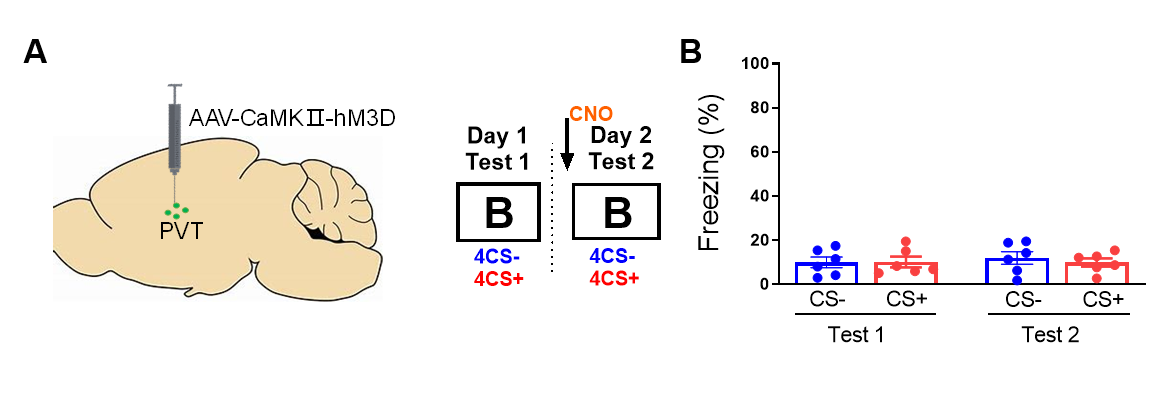


**Fig. S2. Lack of freezing responses in naive animals with PVT stimulation**

(A) Schematic illustration and procedure of chemogenetic stimulation of PVT excitatory neurons.

(B) Freezing levels during CS- and CS+ pre-and-post chemogenetic activation (two-way ANOVA, interaction, F (1,20) = 0.2283, P = 0.6380, stimulus, F (1,20) = 0.1478, P =0.7047, treatment, F (1,20) = 0.1563, P = 0.6967; N = 6 mice, CS-(Test 1) vs. CS-(Test 2), P > 0.9999, CS+ (Test 1) vs. CS+ (Test 2), P > 0.9999, Bonferroni’s post-test).


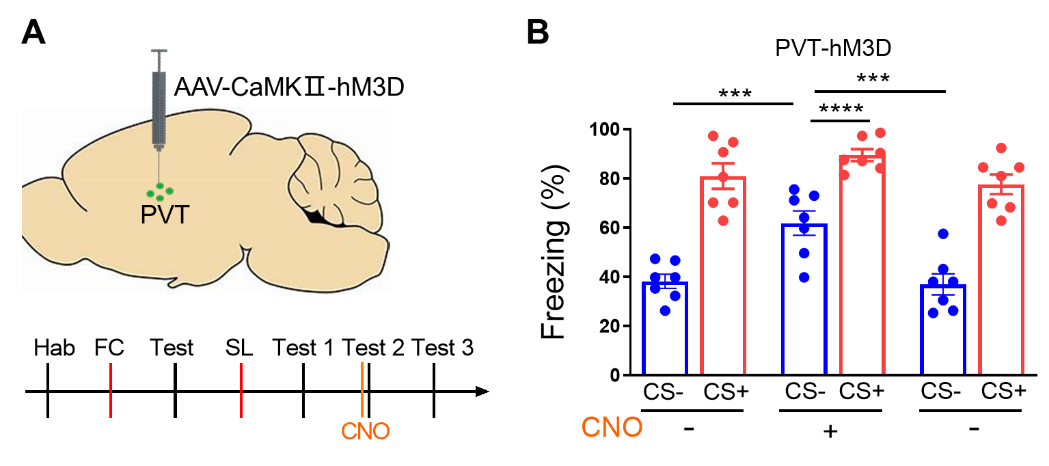


**Fig. S3. Impairment of safety memory expression in female mice with PVT stimulation**

(A) Schematic illustration and procedure of chemogenetic stimulation of PVT excitatory neurons.

(B) Freezing levels during CS− and CS+ post-safety learning（two-way ANOVA, N = 7 mice, CS-(pre CNO) vs. CS-(CNO), P < 0.001, CS-(CNO) vs. (post CNO), P < 0.001, Bonferroni’s post-test）.


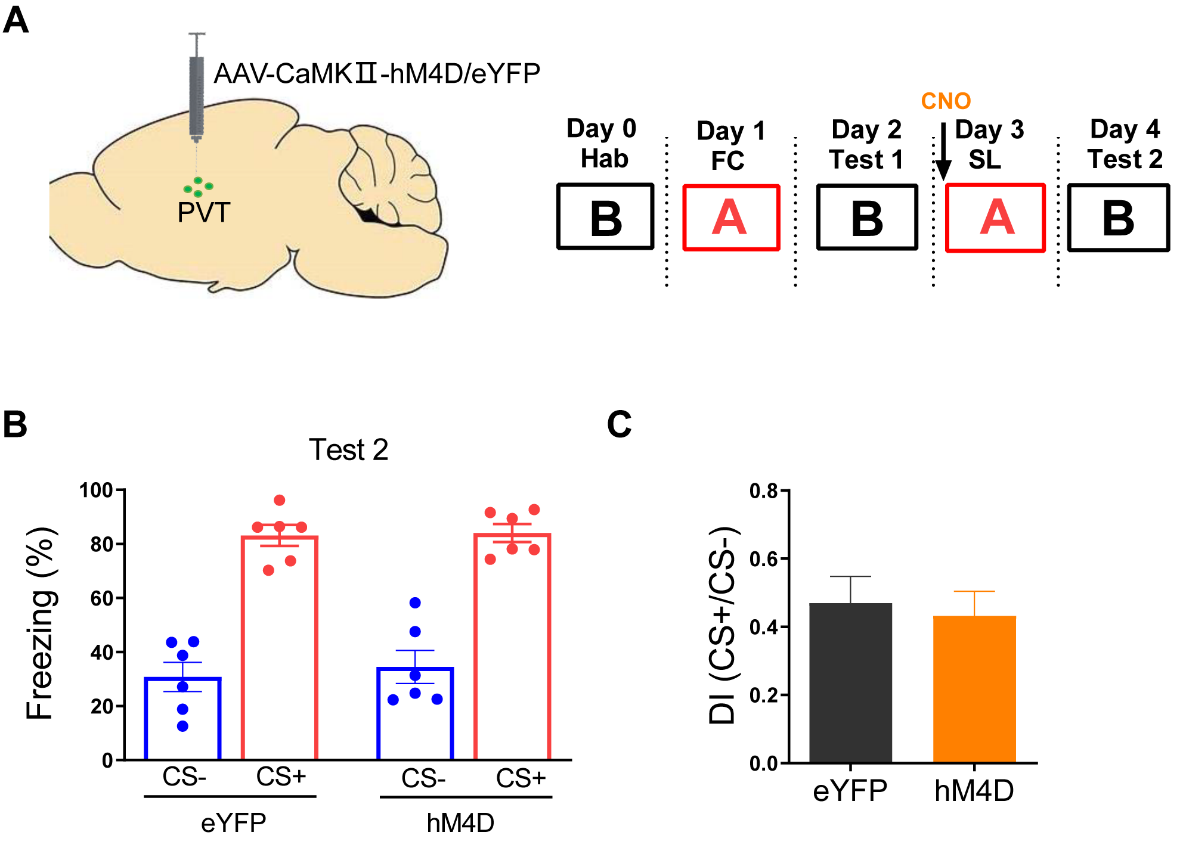


**Fig. S4. Effect of PVT inhibition on safety learning formation**

(A) Schematic illustration and procedure for chemogenetic inhibition of PVT neurons during safety learning.

(B) Freezing levels during CS− and CS+ post-safety learning in mice injected with hM4D virus or control virus (two-way ANOVA, interaction, F (1, 20) = 0.1530, P=0. 6998, stimulus, F (1, 20) = 98.21, P<0.0001, treatment, F (1, 20) = 0.5650, P=0.4610; Control, N =6 mice, hM4D, N = 6 mice, CS-(Control) vs. CS-(hM4D), P=0.9605, CS+(Control) vs. CS+(hM4D), P = 0.6734, Bonferroni’s post-test).

(C) Discrimination index post-safety learning in mice injected with hM4D virus or control virus (two-tailed unpaired t test, t=0.1981, df= 10; Control, N = 6 mice, hM4D, N = 6 mice, Control vs. hM4D, P=0.8470).

**Fig. S5. Quantification of labeled stress-activated neurons in the PVT per** **tissue area (0.16 mm^2^, n = 5 mice).**


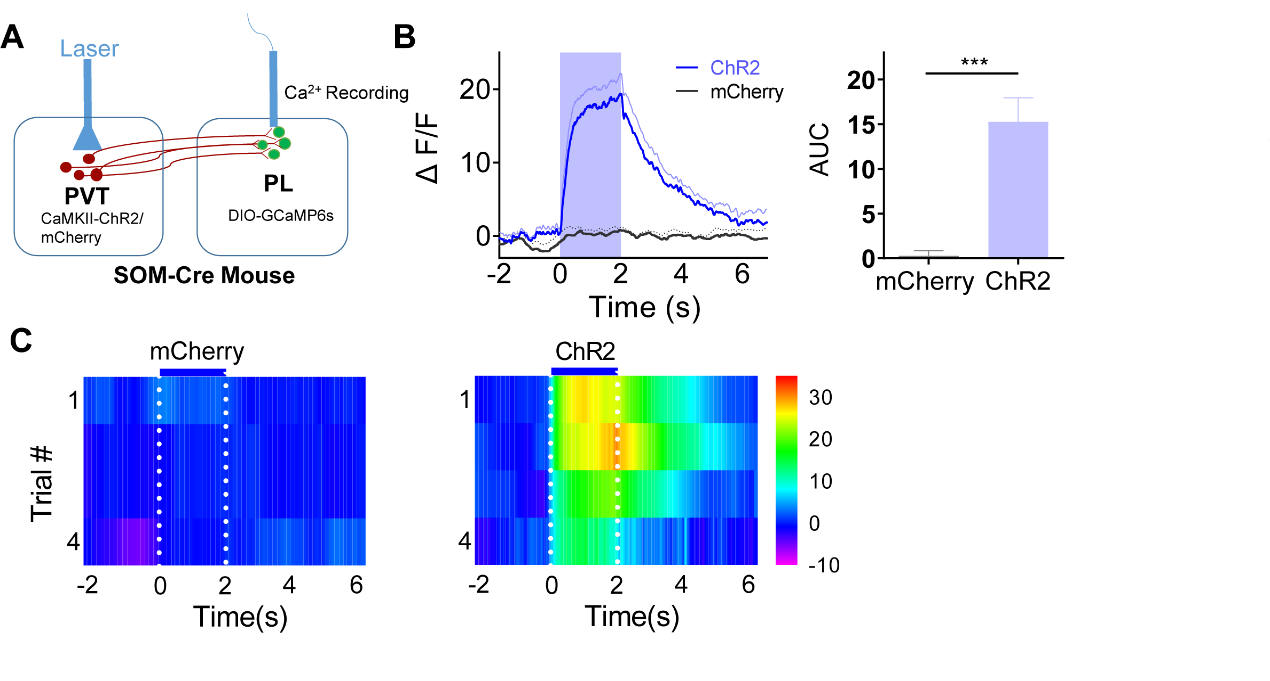


**Fig. S6. PVT stimulation activates PL SOM-INs**

(A) Schematic of the injection of DIO-GCaMP6 virus into the PL and CaMKII-CHR2 virus into the PVT in SOM-Cre mice.

(B) Averaged Ca^2+^ responses(left) in SOM-INs during PVT stimulation in mice injected with either ChR2 or mCherry virus, and quantification of responses (AUC) (right, two-tailed unpaired t test, t = 4.969, df = 9; 5 mice, mCherry; 6 mice, ChR2, mCherry vs. ChR2, P < 0.001).

(C) Heat map of Ca^2+^ responses in SOM-INs during PVT stimulation in mice injected with either ChR2 or mCherry virus.

**Fig. S7. Induction of freezing behavior in naïve animals by optogenetic stimulation of PL SOM-INs**

Freezing levels during baseline and blue light stimulation in mice injected with CHR2 or control virus (two-way ANOVA, Control, N =6 mice, ChR2, N = 5 mice, Base (eYFP) vs. Light (eYFP), P=0.8324, Base (ChR2) vs. Light (ChR2), P = 0.0007, Bonferroni’s post-test).


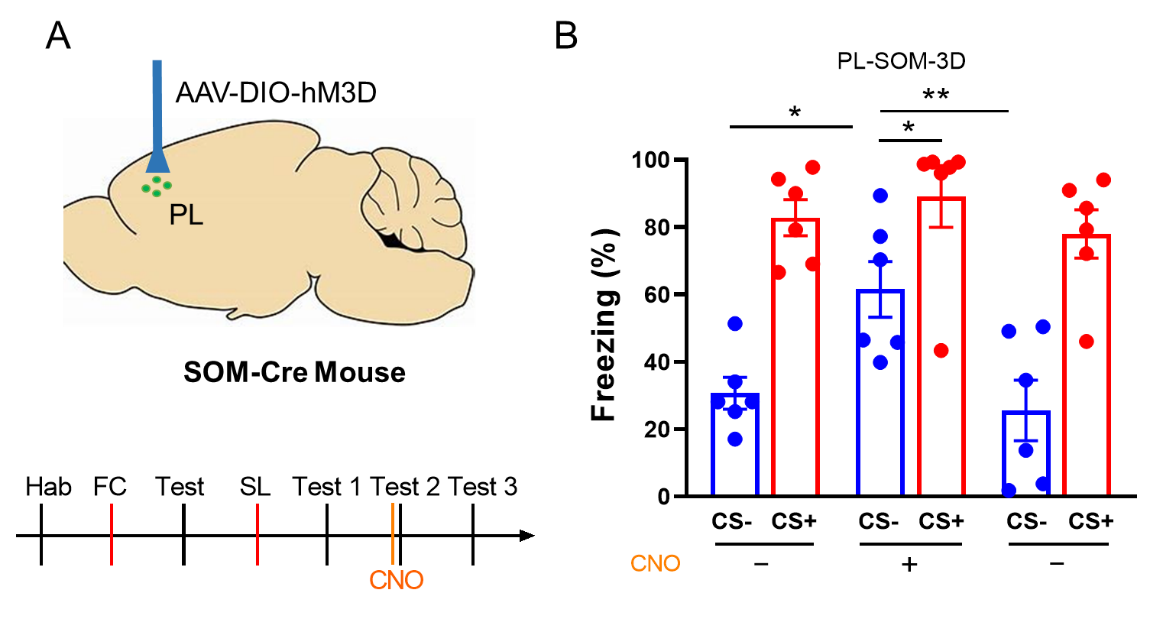


**Fig. S8. Impairment of safety memory expression in female mice through chemogenetic stimulation of PL SOM-INs.**

(A) Schematic illustration and procedure of chemogenetic stimulation of PVT excitatory neurons.

(B) Freezing levels post-SL in mice injected with hM3D virus in SOM-Cre mice (N = 6 mice, CS-(pre CNO) vs. CS-(CNO), P < 0.05, CS-(CNO) vs. CS- (post CNO), P < 0.01).

**
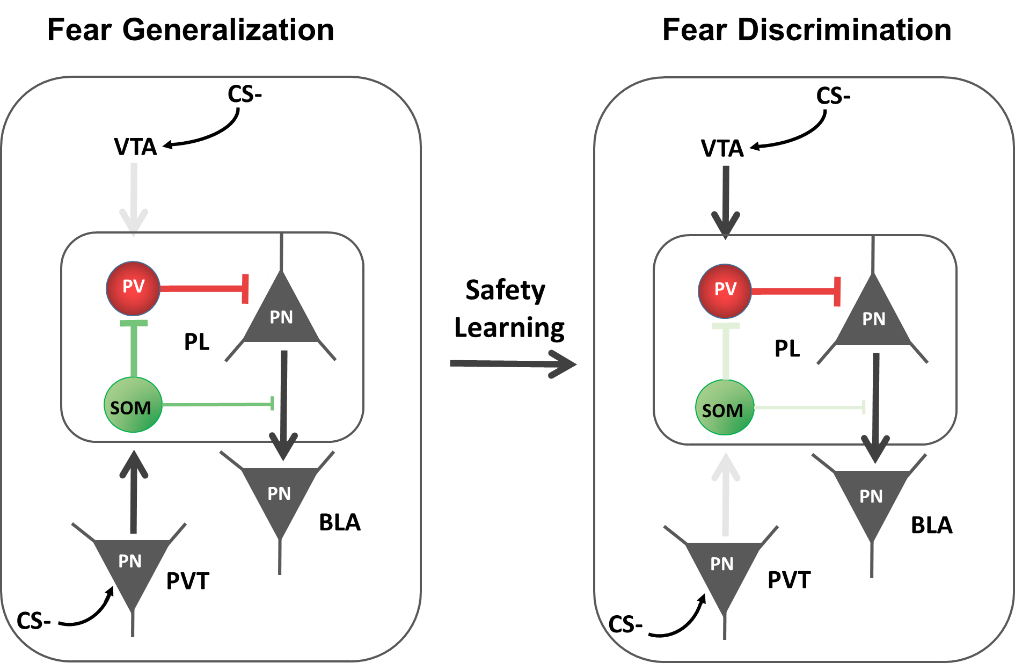
**

**Fig. S9. Model of safety learning circuit mechanism.**

Left: In the fear generalization state, CS- and CS+ generate high activity in the PVT and high fear via outputs to PL SOM-INs, which disinhibit PN via PV-IN inhibition. Disinhibited PN in the PL then promotes fear generalization by activating subcortical regions like the basolateral amygdala (BLA).

Right: In the fear discrimination state, PVT remains inactive upon presentation of CS- (the safety cue), resulting in non-activation of SOM-INs and disinhibition of PV-INs. Disinhibited PV-INs provide potent perisomatic inhibition to PN in the PL, effectively suppressing their outputs and reducing fear expression. Additionally, safety learning potentiates VTA inputs to PL PV-INs. The convergence on the PL PV-INs of the PVT-PL and VTA-PL circuits allows for coordinated modification of fear discrimination.

**Supplementary Table 1. Sample size**

| **Figure** | **Sample size** |
| --- | --- |
| Fig. 1B | n = 7 male for air puff, tail suspension, and foot shock group |
| Fig. 1D-F | n =7 male for behavioral test and PVT recording |
| Fig. 1G-H | n =9 male for behavioral test and PVT recording |
| Fig. 2B | n =6 male for PVT-CaMKII-hM3D group, n =6 male for PVT-CaMKII-eYFP group |
| Fig. 2D | n =6 male for PVT-PL-hM3D group, n =5 male for PVT-PL-eYFP group |
| Fig. 2F | n =7 male for PVT-CaMKII-hM3D group, n=6 for PVT-CaMKII-eYFP group |
| Fig. 2H | n =6 male for PVT-CaMKII-hM3D, PL-PV-hM3D group, n=6 male for PVT- CaMKII-eYFP, PL-PV-mCherry group |
| Fig. 3B-C | n =7 male for behavioral test and PVT recording |
| Fig. 3E | n =6 male for behavioral test |
| Fig. 4D-E | n =6 male for recording of stress-activated neurons in PVT |
| Fig. 4G | n =6 male for behavioral test |
| Fig. 4I | n =7 male for behavioral test |
| Fig. 5B-D | n =6 male for behavioral test and PL SOM-INs recording |
| Fig. 5F | n =6 male for PL-SOM-CHR2 group, n =6 male for PL-SOM-eYFP group |
| Fig. 5H | n =9 male for PVT-hM3D, PL-SOM-hM4D group, n =6 male for PVT-eYFP, PL-SOM-mCherry group |
| Fig. S1 | n =7 male for behavioral test |
| Fig. S2 | n =6 male for PVT-CaMKII-hM3D group |
| Fig. S3 | n=7 female for PVT-CaMKII-hM3D group |
| Fig. S4 | n =6 male for PVT-CaMKII-hM4D group, n =6 male for PVT-CaMKII-eYFP group |
| Fig. S5 | n =5 male for quantification |
| Fig. S6 | n =5 male for PVT-ChR2, PL-SOM-GCaMPs group, n =6 male for PVT-mCherry, PL-SOM-GCaMPs group |
| Fig. S7 | n =5 male for PL-SOM-CHR2 group, n =6 male for PL-SOM-eYFP group |
| Fig. S8 | n =6 female for PL-SOM-hM3D group |
